# Grain Refinement and Aging Hardening of the Mg-10Gd-3Y-2Ag-0.4Zr Alloy Produced by a Two-Step Forming Process

**DOI:** 10.3390/ma11050757

**Published:** 2018-05-09

**Authors:** Huiyan Ning, Yandong Yu, Bo Gao, Lirong Xiao, Lihua Wen, Zehua Yan, Li Li, Xuefei Chen

**Affiliations:** 1 School of Materials Science and Engineering, Harbin University of Science and Technology, Harbin 150040, China; ninghuiyan2000@163.com (H.N.); yanzehua5290@163.com (Z.Y.); 2 School of Mechanical and Electrical Engineering, Heilongjiang Institute of Technology, Harbin 150050, China; wenlihua518@126.com; 3 Nano and Heterogeneous Material Center, School of Materials Science and Engineering, Nanjing University of Science and Technology, Nanjing 210094, China; gaobo@njust.edu.cn (B.G.); lili_smse@njust.edu.cn (L.L.); 4 Institute of Microstructure and Property of Advanced Materials, Beijing University of Technology, Beijing 100124, China; xiaolr620@126.com; 5 State Key Laboratory of Nonlinear Mechanics, Institute of Mechanics, Chinese Academy of Sciences, Beijing 100190, China

**Keywords:** Mg-Gd-Y-Ag-Zr alloy, rolling, thermal treatment, microstructure, mechanical property

## Abstract

Grain refinement and precipitation are two effective ways to improve the mechanical properties of Mg-RE alloys. In this work, a two-step forming process is proposed. This includes cold rolling and subsequent annealing at high temperature for a short duration. By the two-step forming process, grains can be refined from 100 μm to 20 μm in compare with ~30 μm by common hot rolling at 450 °C for a reduction of 80%. The sample shows more distinct aging hardening, as the hardness amplification of 60 HV is twice that of the hot-rolled sample. The precipitation is observed by high angle annular dark field-scanning transmission electron microscopy (HAADF-STEM). Dynamic precipitation in the sample by the two-step route is found to be effectively suppressed. Interestingly, after subsequent annealing, the density of precipitation, especially β′, become much higher than that in hot-rolled samples.

## 1. Introduction

Magnesium alloys have been widely applied in the automotive and aerospace industries due to their lightweight, high specific strength and good shielding effect (the capacity of reflection and/or absorption of electromagnetic radiations by a material) [1]. However, some applications are limited due to their relatively poor mechanical properties, especially in elevated temperatures (~200 °C), compared to Al alloys [2]. Rare earth elements (RE) play particular roles in metallurgy and materials due to their unique configuration of an extra-nuclear electron [3]. The addition of rare earth elements to magnesium can improve the strength, thermal stability and corrosion resistance effectively [4,5]. Gadolinium exhibits a large solubility in Mg-Gd alloys at elevated temperature, and it drops following the exponential relation with decreasing temperature [6]. The large amount of precipitation occurring in Mg-Gd alloys by aging results in the markedly high strength. Thus, Mg-Gd alloys can be strengthened through both grain refinement and aging. Tremendous attention has been attracted to the aging hardening behavior in Mg alloys [7,8,9].

Recently, much attention has been paid to the study of Mg-Gd alloys, since the mechanical properties of Mg-Gd alloys can be greatly improved via altering the precipitation by arranging an appropriate chemical composition, plastic deformation and heat treatment [10]. Mg_5_Gd is known to be the dominant phase to strengthen the Mg-Gd alloys [11]. It is reported that the strength of Mg-Gd-Y-Zr alloys has been improved by various routes combining plastic deformation and thermal treatment [10,12,13,14,15]. In addition, the long-period stacking order (LPSO) has attracted more attention to investigate Zn in Mg-Gd alloys. Zheng et al. [16], Yamasaki et al. [17], and Honma et al. [18] proposed that the LPSO structure and texture variation was the key factor to improve the mechanical properties of Mg alloys, according to the fiber structure of the matrix after aging. Interestingly, the addition of Ag to Mg-Gd alloys might induce new precipitation excluding β [19,20,21]. The addition of Ag can also promote the segregation of alloy elements in twin boundaries, stacking faults and grain boundaries. Consequently, the addition of Ag can improve the strength-ductility synergy and decrease the duration of aging [19]. The yield strength and ultimate tensile strength of Mg-8.5Gd-2.3Y-1.8Ag after solution and aging treatment has been reported to reach 268 MPa and 403 MPa, respectively [22].

Since grains are refined to sub-micro or nano scale with high angle grain boundaries by severe plastic deformation (SPD), the mechanical properties of Mg alloys are remarkably improved. It is therefore interesting to study the effect of the precipitation hardening of RE on the strain hardening of Mg alloys after SPD. Zhou et al. [23] and Reza Alizadeh et al. [24] reported that mechanical properties of Mg-Gd alloys can be enhanced by SPD for grain refinement. Moreover, Xiao et al. [25] and Hou et al. [26] found that more notable precipitation hardening at T6 (the complete artificial aging after solution) than T5 (the incomplete artificial aging after solution) after hot deformation in Mg-Gd alloys. They also proposed that the density of precipitation was reduced by the dynamic precipitation during hot deformation. This means that precipitation hardening was weakened in the aging condition. As a result, it is crucial to develop a suitable processing route for strain aging hardening of RE. In this work, the Mg-10%Gd-3%Y-2%Ag-0.4Zr is investigated to develop an appropriate processing route. The microstructure and mechanical properties of Mg-Gd alloys by various rolling methods and thermal treatments are studied, and the strengthening mechanism of Mg-Gd alloys are systematically analyzed. 

## 2. Experimental Producer

The composition of the alloy is Mg-10Gd-3Y-2Ag-0.4Zr (GWQ1032K) (wt %) (Table 1), which was determined by an inductively coupled plasma atomic emission spectroscopy (ICP-AES) analyzer (PerkinElmer, Plasma 400). The ingot was prepared by melting high purity Mg and Ag (99.95%, wt %) and master alloys of Mg-25Gd (wt %), Mg-25Y (wt %) and Mg-30Zr (wt %) in a mild steel crucible at approximately 750 °C. The melting was performed under a mixed flowing protective atmosphere of CO_2_ and SF_6_ with a volume ratio of 100:1. The melt was poured into a steel mold which was pre-heated to 200 °C [27]. The as-cast ingot with a size of 20 cm × 30 cm × 2 cm was homogenized at 500 °C for 12 h and quenched to room temperature in water (T4 treatment) [27]. The T4-treated samples were cut into plates with a dimension of 30 mm × 20 mm × 2 mm. Then, the plates were rolled at 400 °C, 450 °C and 530 °C for 20%, 40%, 60% and 80% with rolling speed of 22 mm/s. The thickness reduction between every two passes is 0.1 mm. Before hot rolling, the solution treated slabs were preheated at corresponding temperature for 30 min in the resistance furnace. The specimen rolled at 450 °C with thickness reduction of 80%. Then the specimen was isothermal aged at 180 °C, 200 °C, 225 °C and 250 °C for a duration ranging from 0.25 h to 256 h in an oil bath. Annealing at 530 °C for 20 s was carried out on the sample rolled at room temperature and then followed by the isothermal aging treatment at 180 °C for a duration ranging from 0.25 h to 256 h.

The specimens for characterization were ground using 320, 600 and 800 grit sand papers and then polished on a woolen cloth using a 1 μm diamond suspension. Samples were further polished on polishing cloth using magnesia suspension to a mirror finish. To determine the grain size under optical microscope, samples were etched by a lab-prepared solution with a recipe of 100 mL ethyl alcohol with 5 g picric acid and 5 g acetic acid. The X-ray diffraction (XRD) analysis was performed on a Pan Analytical X″ Pert diffractometer (X′Pert PRO, PANalytical B.V, Holland) employing Cu Kα radiation at 50 kV and 40 mA. Transmission electron microscopy (TEM) specimens were cut from the plate and gently polished to a thickness of ~25 μm. Perforation by ion milling was carried out on a cold stage (~50 °C) with low angle (<3.5°) and low energy ion beam (<3 keV). The bright-field TEM images were obtained from a JEOL-2010F microscope operating at 200 kV. Vickers microhardness tests of the specimens were performed under 100 gf (0.98 Newton) loading for 15 s at a MATSUZAWA-VMT-7S tester (VMT-7S, MATSUZAWA, Japan). At least 10 indentations were used to calculate the mean value and standard deviation.

## 3. Results and Discussion

**Figure 1:**
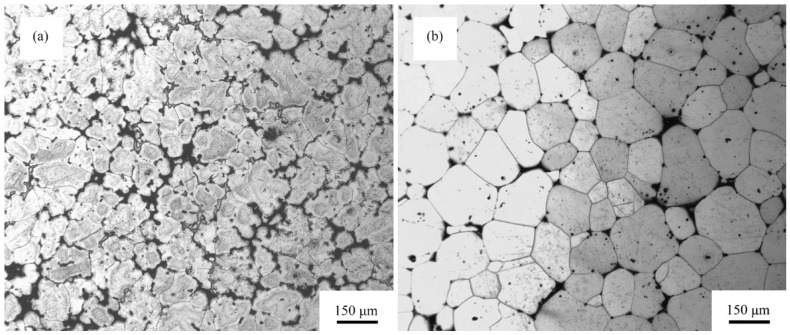
Optical microstructure images of GWQ1032K alloy: (**a**) as-cast; (**b**) solid solute (T4).

As Figure 1a shows, the initial grain size of as-cast GWQ1032K alloy is ~100 μm. The morphology of the ingot consists of α-Mg matrix and eutectic phase crystallizing at the irregular-shaped grain boundaries. The brittle eutectic phase forming after casting generally induces the crack initiation source during plastic deformation [28]. Thus, the eutectic phase is the key factor for the poor ductility and formability of casted Mg-Gd-RE alloys. In order to eliminate the influence of eutectic phase and enhance the aging hardening, solution treatment is necessary for Mg alloys before deformation. The typical microstructure of GWQ1032K alloy after solution treatment at 500 °C for 12 h is shown in Figure 1b. Obviously, the eutectic structure in the ingot has disappeared after solution treatment. The grains are generally equiaxed and about ~100 μm with straight grain boundaries. However, some discontinuous second phase can still be observed in the matrix, because they are difficult to dissolve totally by solution.

**Figure 2:**
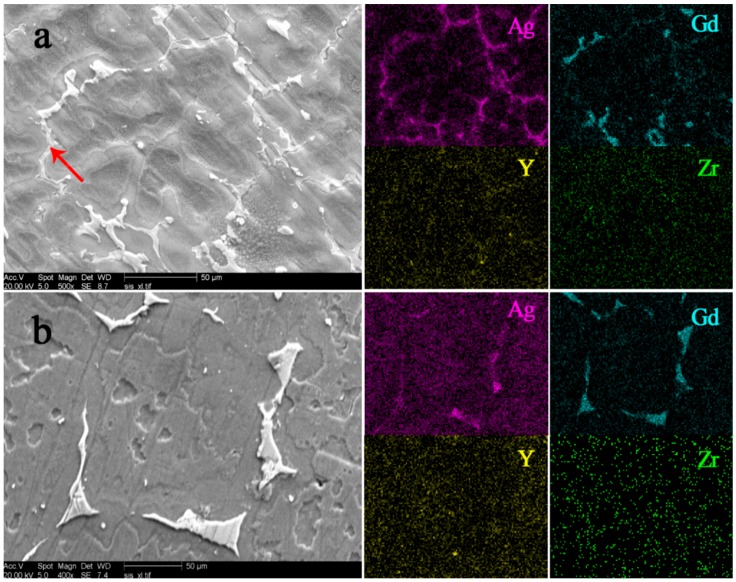
SEM and EDS mapping images of GWQ1032K alloy: (**a**) as-cast; (**b**) solid solute (T4).

The evolution of precipitation in grain boundaries for the GWQ1032K alloy is investigated. As shown in Figure 2a, a large amount of continuous precipitation distributes at the grain boundary in ingot. The results of EDS indicate that the precipitation is mainly composed of Ag and Gd without Y or Zr [19]. After solution treatment at 500 °C for 12 h, only some discontinuous precipitation can be observed at the grain boundaries (Figure 2b). It is revealed that Ag has been solute into the matrix after solution treatment, and the residual precipitation consists of Gd without Y and Zr. 

**Figure 3:**
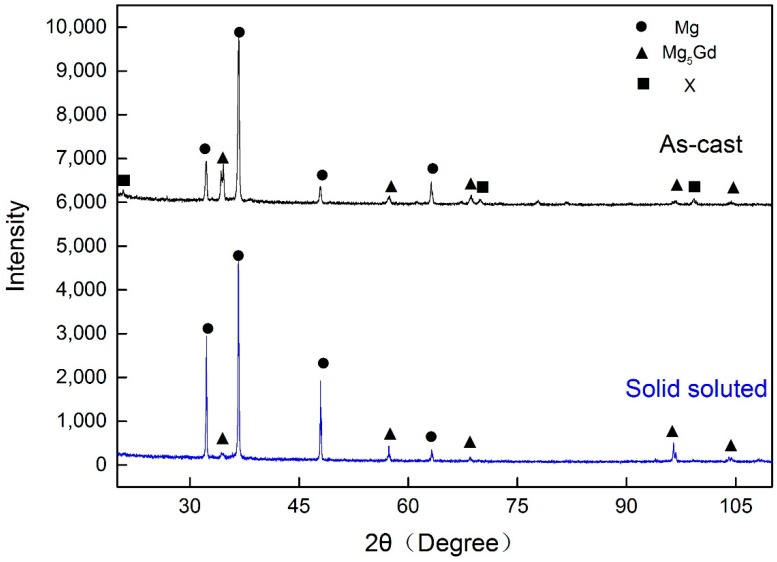
XRD curves of as-cast and solid solute (T4) alloys.

Figure 3 shows the XRD patterns of as-cast and solid solute samples to reveal the phase compositions in the alloys. The peaks marked by black triangles represent the well-known Mg_5_Gd phase which exists in most casting Mg-Gd system alloys [29]. Moreover, another phase marked by the black squares is an Ag-enriched phase which is uncommon in cast Mg-Gd alloys. The composition of this phase is estimated by EDS in SEM, as marked by the red arrow in Figure 2a. This indicates that the ratio of Mg and RE in the phase is around 17:3, which is similar to the one in Mg-Gg-Ag alloys reported in [30]. After solid solution, most of the Mg_17_RE_3_ phase dissolves into the matrix, while the Mg_5_Gd phase remains in grain boundaries. This reveals that Ag containing phase is much easier to solute during heating.

**Figure 4:**
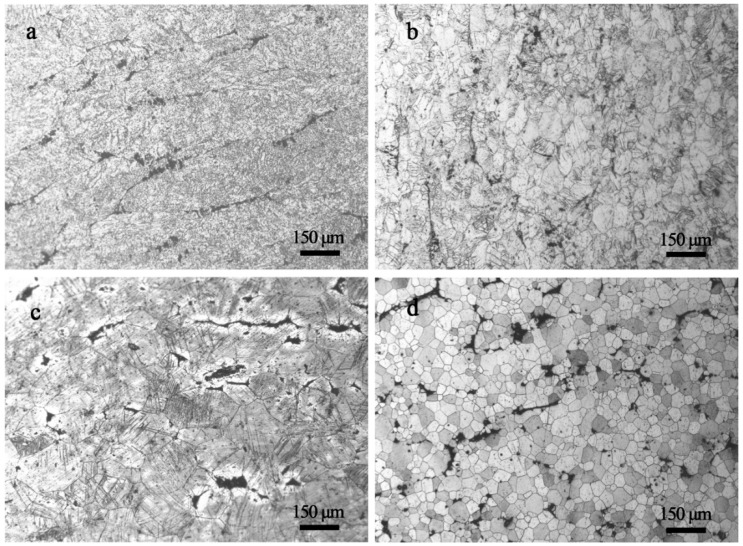
Optical microstructures of GWQ1032K alloys by hot rolled with reduction of 80%: (**a**) 400 °C; (**b**) 450 °C; (**c**) 500 °C; (**d**) sample prepared by the two-step forming process.

By introducing a high density of grain boundaries to hinder dislocation motion, grain refinement is one of the effective methods to strengthen materials [31]. In addition, the poor symmetry of hexagonal close-packed structure (HCP) of Mg alloys results in inhomogeneous deformation between grains with different orientations during plastic deformation [32]. However, this can be weakened through grain refinement. Thus, grain refinement by plastic deformation is one of the common ways to improve both strength and ductility for Mg alloys [33]. Figure 4a–c shows the microstructures of GWQ1032K alloy rolled with a reduction of 80% at 400 °C, 450 °C and 500 °C. It is indicated that the deformation temperature has an apparent effect on grain refinement. Figure 4a shows that the grains elongate along the rolling direction without obvious recrystallization at 400 °C. A high density of defects also can be observed inside grains. At 450 °C, recrystallization occurs, and the grain is refined to ~30 μm. Many laminate structures similar to deformed twins can be found in the grains [34], as Figure 4b shows. However, the grains grow obviously at 500 °C (Figure 4c) due to the rapid dynamic recovery. Consequently, the grain refinement for hot-rolled GWQ1032K alloy is closely related to the deformation temperature. In order to utilize dynamic recrystallization and control the rate of dynamic recovery, the suitable rolling temperature slightly over the critical recrystallization temperature is the key factor. Thus, in this work, a novel two-step route including rolling at room temperature and subsequently annealing at elevated temperature is studied. The Mg alloy was firstly rolled with thickness reduction of 40% and then followed by annealing at 530 °C for 20 s. The optical micrograph of the Mg alloy by the new route is shown in Figure 4d. The grains are smaller (with an average grain size of ~20 μm) compared to that of hot rolled at 450 °C. In addition, the density of deformed twins is reduced, and this is beneficial to further deformation to obtain a good strength-ductility synergy [35].

**Figure 5:**
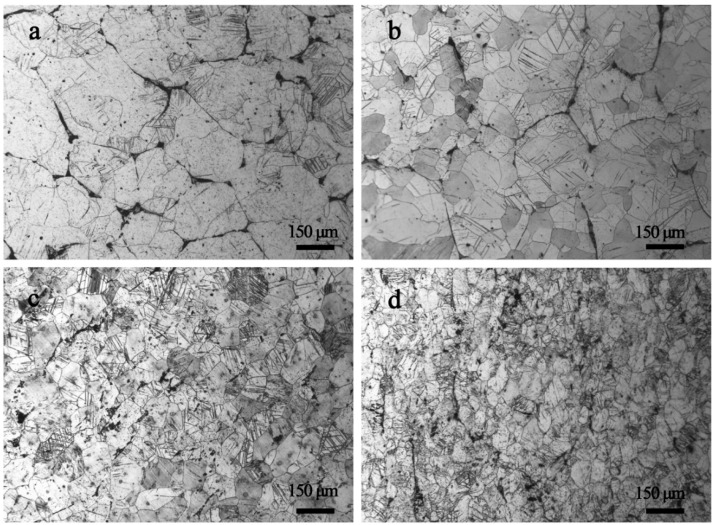
Optical microstructure of GWQ1032K alloy prepared by hot rolling at 450 °C with reduction of: (**a**) 20%; (**b**) 40%; (**c**) 60%; (**d**) 80%.

Figure 5 shows the microstructure of GWQ1032K alloy rolled at 450 °C with various total reductions. With a reduction of 20%, the deformed grains are slightly elongated, and some deformed twins intersect with each other as Figure 5a shows. As shown in Figure 5b, the recrystallization occurs in the coarse grain after 40% rolling, and the recrystallization grain size is about 30–50 μm. With the increasing of rolling reduction to 60%, the grains become homogeneous recrystallized grains with many deformation twins (Figure 5c). Finally, the grains are refined from 150 μm to 30 μm after rolling with reduction of 80% (Figure 5d).

**Figure 6:**
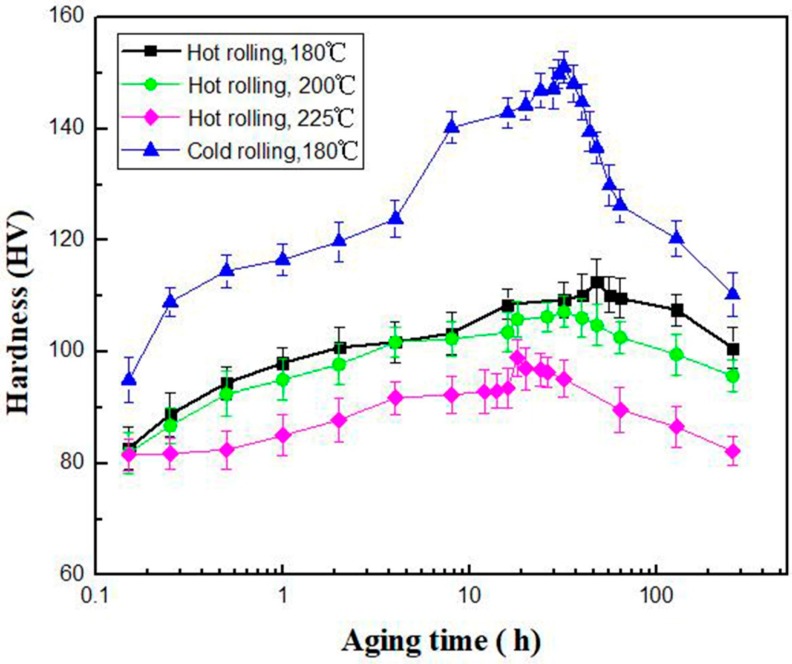
Hardness evolution of GWQ1032K alloy aged at 180 °C, 200 °C and 225 °C.

Precipitation strengthening is another effective way to improve the mechanical properties of Mg alloys. It is known that the second phase after aging in nano-scale is effective in blocking dislocation motion [36,37]. Figure 6 shows the hardness evolution after aging treatment at 180 °C, 200 °C and 225 °C, respectively, for GWQ1032K alloy rolled at 450 °C with 80% reduction. It is revealed that the aging hardening at 180 °C is the most prominent, with the peak at 48 h. The aging hardening is weakened with the increase of aging temperature. At the same time, the aging peak has a tendency for earlier shift. The peak time is reduced about ~20 h from 225 °C to 180 °C. In general, for the hot-rolled GWQ1032K alloy, aging hardening ability is limited with a hardness amplification within 30 HV. However, distinct aging hardening is found in sample prepared by the two-step route when aging at 180 °C. The hardness increases from 95 HV to 150 HV with the amplification as twice as that of hot-rolled Mg alloys. Meanwhile, the peak holding time is just 32 h. The increase of 40 HV in peak hardness may attributed to the following two factors: (a) the increase of 10 HV is due to grain refinement from recrystallization; (b) the extra 30 HV is due to aging hardening behaviors.

**Figure 7:**
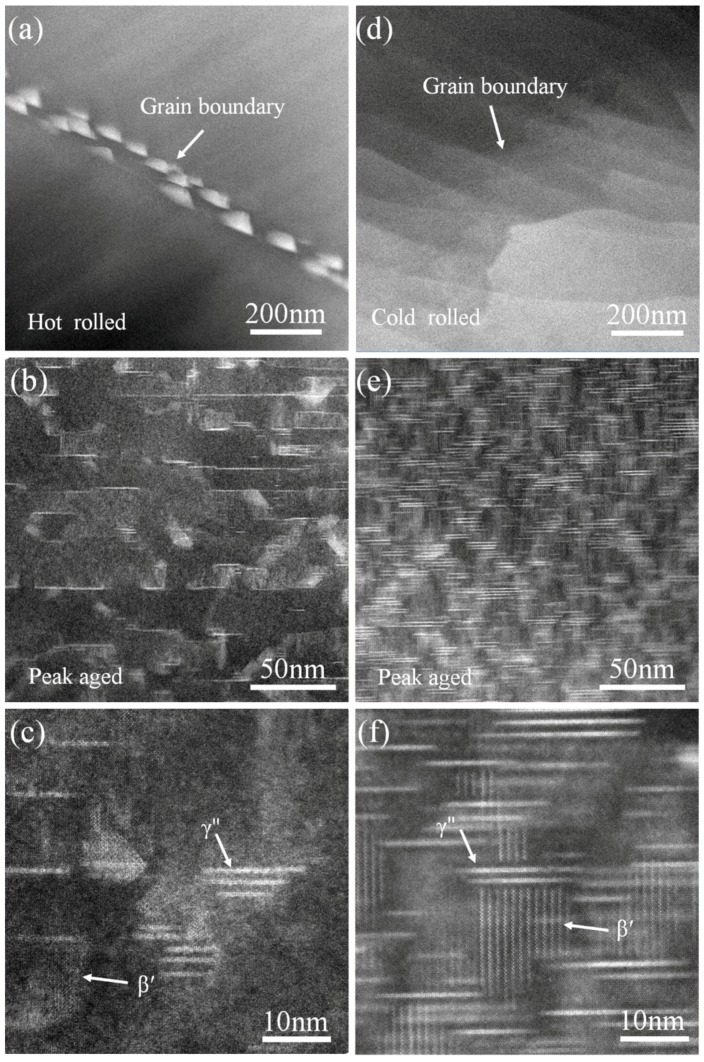
High angle annular dark field-scanning transmission electron microscopy (HAADF-STEM) images of GWQ1032K alloy: (**a**) hot rolling at 450 °C with 80% reduction; (**b**,**c**) aging at 180 °C with 48 h for hot rolled sample; (**d**) sample prepared by the two-step forming process; (**e**,**f**) aging at 180 °C with 32 h for sample prepared by the two-step forming process.

Since the atomic number of RE is higher than Mg, Z-contrast can be clearly observed by high angle annular dark field-scanning transmission electron microscopy (HAADF-STEM) at the RE-rich locations. Figure 7a shows a large amount of high density second phase with RE-rich precipitates along the grain boundaries in the hot-rolled sample. The average size of the precipitation is ~40 nm. The nano-scale precipitation results from the dynamic precipitation of the supersaturated GWQ1032K alloy during hot rolling [26,38]. The segregation of RE at grain boundary is mainly attributed to the following two factors: firstly, defects such as dislocation tend to accumulate at the grain boundary in deformed samples [31,39]; secondly, the grain boundary shows a more severe lattice mismatch and higher stored energy than the interior [40]. The segregation decreases the supersaturation of RE in matrix and influences subsequent precipitation. For GWQ1032K alloy prepared by cold rolling and subsequent annealing with short duration, clean grains and boundaries are shown without dynamic precipitation (Figure 7d). This further confirms that grain refinement mainly contributes to the increase of hardness. A large amount of second phase precipitates both in grains of hot-rolled and cold-rolled samples are observed after aging treatment at 180 °C. As Figure 7b and e shows, under the same aging condition, precipitation density is observed to be higher in samples prepared by cold rolling and annealing than that of hot-rolled samples. This is due to the element loss induced by dynamic precipitation in hot-rolled Mg alloy. As Figure 7c,f shows, two kinds of precipitation are observed, which are typical cylindrical β′ and basal γ″ precipitation, respectively (Figure 7c,f). The co-existent β′ and γ″ precipitation can effectively suppress basal and cylindrical slip to strengthen GWQ1032K alloy. The β′ precipitation is closely related to Gd, and the γ″ precipitation is mainly introduced by Ag [41,42]. Figure 7c,f further indicates the higher density of β′ precipitation in the sample prepared by cold rolling and annealing results in the more notable aging hardening. The loss of Gd induced by dynamic precipitation for hot-rolled sample results in the less density of β′ precipitation.

## 4. Conclusions

An effective two-step forming process that combines cold rolling and subsequent annealing at high temperature for a short duration is provided for Mg-RE alloys in the present study. In comparison with common hot rolling, finer grains can be obtained through this novel route. Combining with subsequent aging treatment, Mg-RE alloys can be further strengthened, because this processing route can effectively suppress the dynamic precipitation of RE which maintains superior aging hardening ability. In conclusion:

(1) For Mg-10Gd-3Y-2Ag (GWQ1032K) alloy prepared by cold rolling with 40% reduction and subsequent annealing at 530 °C for 20 s, the grains are refined from 100 μm to 20 μm, which exhibits more effective grain refinement compared with common hot rolling at 450 °C in a thickness reduction of 80% (with refined grains ~30 μm). The hardness results reveal a higher value of 95 HV for Mg alloy fabricated by the two-step forming process than that of the hot-rolled sample (80 HV).
(2) The sample prepared by the two-step forming process shows more distinct aging hardening, with the hardness amplification of 60 HV, which is twice that of the hot-rolled sample. As a result of the effective suppression of dynamic precipitation by the two-step forming process, the density of precipitation at grain boundary is far less than that in the hot-rolled sample, and after aging, the density of precipitation for Mg alloy from the two-step processing route, in particular of β′ precipitation, is much higher than hot-rolled Mg alloy.

## Figures and Tables

**Table 1:** Chemical composition of original alloy Mg-10Gd-3Y-2Ag-0.4Zr.

Element	Gd	Y	Ag	Zr	Mg
Content wt %	10	3	2	0.4	84.6
